# The Prevalence of Tobacco Use at Federally Qualified Health Centers in the United States, 2013

**DOI:** 10.5888/pcd14.160510

**Published:** 2017-04-06

**Authors:** Susan A. Flocke, Richard Hoffman, Jan M. Eberth, Hyunyong Park, Genevieve Birkby, Erika Trapl, Steve Zeliadt

**Affiliations:** 1 Department of Family Medicine & Community Health, Case Western Reserve University, Cleveland Ohio; 2 Department of Epidemiology & Biostatistics, Case Western Reserve University, Cleveland Ohio; 3 Prevention Research Center for Healthy Neighborhoods, Case Western Reserve University, Cleveland Ohio; 4 Case Comprehensive Cancer Center, Cleveland, Ohio; 5 Division of General Internal Medicine, University of Iowa Carver College of Medicine, Iowa City, Iowa; Holden Comprehensive Cancer Center, University of Iowa, Iowa City, Iowa; 6 South Carolina Rural Health Research Center, University of South Carolina, Columbia, South Carolina; Department of Epidemiology and Biostatistics, Arnold School of Public Health, University of South Carolina, Columbia, South Carolina; Cancer Prevention and Control Program, University of South Carolina, Columbia, South Carolina; 7 University of Washington, School of Public Health, Seattle, Washington.

## Abstract

We explored tobacco use across federally qualified health centers (FQHCs) and compared data on state-level tobacco use between FQHC patients and the general population. We used data from the Uniform Data System (UDS) and the Behavioral Risk Factor Surveillance System (BRFSS) to generate estimates of 2013 prevalence of tobacco use among adults aged 18 years or older. According to UDS data, the overall prevalence of tobacco use was 25.8% in FQHCs compared with 20.6% in the general population represented by BRFSS data, an average of 5.2 percentage points (range, −4.9 to 20.9) higher among FQHCs. Among FQHCs, the burden of tobacco use and the opportunity for offering cessation assistance is substantial.

## Objective

Tobacco use contributes to substantial illness and death in the United States (1). Although prevalence of tobacco use has declined during the past decade among some demographic groups, rates have remained steady and even increased among some socially and economically disadvantaged populations (2).

Federally qualified health centers (FQHCs), which provide comprehensive health services to economically disadvantaged populations in rural and urban communities in the United States, are required to collect data on tobacco use screening and tobacco cessation counseling rates as Uniform Data System (UDS) measures. Understanding rates of tobacco use among FQHC clients can guide efforts to provide resources for tobacco cessation assistance where they are most needed (3–6).

Our study explores differences in tobacco use among FQHCs and compares state-level tobacco use between FQHC patients and the general population.

## Methods

We used 2013 UDS FHQC data, which include quality-of-care indicators and patient demographics, to estimate tobacco use. We included only those FQHCs (967 of 1,202) that obtain tobacco use data from an electronic health record (EHR). Our denominator was the number of adults (≥18 y) having 1 or more medical visits to a FQHC in 2013. The numerator was the number of adults using any form of tobacco including cigarettes, cigars, and smokeless tobacco, as documented during routine patient care.

We estimated the prevalence of adult tobacco users in each state’s FQHC population by summing the total number of tobacco users across FQHCs and dividing by the total number of adult FQHC patients. We also estimated the prevalence of tobacco use for each FQHC and calculated the median and lowest and highest values for FQHCs in each state.

We then compared data on state-level estimates of FQHC tobacco use with data from the 2013 Behavioral Risk Factor Surveillance System (BRFSS), a random-digit–dial telephone survey that collects data on population-level prevalence of health risk behaviors among US adults aged 18 years or older. Three BRFSS items are used to indicate tobacco use: 1) “Have you smoked at least 100 cigarettes in your entire life?”, 2) “Do you now smoke cigarettes every day, some days, or not at all?”, and 3) “Do you currently use chewing tobacco, snuff, or snus every day, some days, or not at all?” Survey participants that responded yes to question 1 and every day or some days to either question 2 or question 3 were identified as tobacco users (7). We applied BRFSS sampling weights to estimate state-specific prevalence of tobacco use (8).

## Results

In total, 1,202 FQHCs reported 2013 UDS data; 967 (80.4%) collected EHR-based tobacco use data. In this subset, the nearly 9 million adult patients seen were similar to the US population in percentage female (58.7%) and Hispanic (16.4%). FQHC patients were less likely than the US population to be older than 65 years (7.3% vs 14.1%) and more likely to be black (20.5% vs 13.0%) or other race (23.6% vs 8.6%). As expected, FQHC patients were more likely to be below the federal poverty level (71.7% vs 14.8%) and uninsured (34.8% vs 13.4%) or using government health insurance (50.8% vs 34.3%).

The overall proportion of tobacco use in FHQCs was 25.8%, and median prevalence was 29.3%, ranging from 0.4% to 94.4% across states (Table). BRFSS data from 2013 estimated US tobacco use at 20.6%, ranging from 12.1% to 30.8% across states.

**Table:** Prevalence of Tobacco Use Among Patients at Federally Qualified Health Centers (N = 967) and a Comparison With Population Prevalence, by State, United States, 2013^a^

State	No. of FQHCs	No. of Adult FQHC Patients	No. of FQHC Patients That Use Tobacco	FQHC Tobacco Use, %		Tobacco Use in Population^c^, %	Percentage Point Difference^d^
				Total^b^	Median (Range)		
All	967	8,762,429	2,258,335	25.8	29.3 (0.4–94.4)	20.6	5.2
Montana	16	53,930	24,611	45.6	40.2 (19.1–77.0)	24.7	20.9
Missouri	18	154,137	62,359	40.5	40.7 (17.2–60.3)	25.8	14.7
Nevada	3	26,250	10,565	40.2	33.0 (23.7–48.9)	21.5	18.7
Michigan	25	237,769	95,341	40.1	40.9 (18.7–63.9)	23.4	16.7
Arkansas	11	79,770	31,742	39.8	41.0 (21.3–82.3)	30.5	9.3
Iowa	11	58,802	23,342	39.7	35.5 (18.6–50.4)	22.9	16.8
South Dakota	6	26,570	10,446	39.3	36.5 (24.7–52.2)	24.3	15.0
Kansas	15	65,335	25,090	38.4	36.9 (16.1–47.7)	23.8	14.6
Indiana	18	157,991	60,588	38.3	36.6 (4.6–57.5)	25.0	13.3
Wyoming	3	8,585	3,088	36.0	36.9 (21.8–53.0)	26.8	9.2
Oklahoma	17	76,648	27,089	35.3	37.6 (14.8–58.4)	28.2	7.1
Ohio	30	209,899	72,597	34.6	38.4 (12.3–72.8)	26.0	8.6
North Dakota	4	15,363	5,293	34.5	33.8 (28.8–38.7)	26.4	8.1
Wisconsin	15	82,817	27,467	33.2	34.9 (9.0–55.5)	21.5	11.7
Tennessee	24	195,473	64,589	33.0	31.9 (9.1–60.1)	27.6	5.4
Louisiana	22	116,474	38,211	32.8	33.6 (13.2–45.6)	27.6	5.2
Alaska	21	43,399	13,873	32.0	33.7 (8.0–68.3)	27.3	4.7
Oregon	26	156,608	48,782	31.1	34.8 (11.4–67.5)	20.3	10.8
Connecticut	9	73,298	21,852	29.8	29.7 (15.2–38.9)	16.7	13.1
Colorado	12	155,570	46,297	29.8	31.7 (19.3–46.7)	20.5	9.3
West Virginia	22	186,695	55,185	29.6	31.1 (5.0–46.8)	34.3	−4.7
Kentucky	16	126,970	37,361	29.4	33.8 (12.7–66.1)	30.8	−1.4
Nebraska	5	26,101	7,675	29.4	34.2 (18.9–44.9)	22.0	7.4
Washington	21	377,869	109,186	28.9	30.2 (9.4–43.1)	18.3	10.6
New Mexico	13	107,381	30,974	28.8	30.5 (16.7–86.1)	21.7	7.1
Maine	15	106,992	30,777	28.8	29.8 (9.6–50.5)	21.5	7.3
South Carolina	14	132,501	38,054	28.7	30.7 (6.4–42.9)	25.0	3.7
New Hampshire	10	47,455	13,566	28.6	37.0 (18.2–73.0)	18.0	10.6
Idaho	8	47,127	13,250	28.1	27.4 (10.2–41.9)	20.5	7.6
Alabama	11	155,704	41,945	26.9	30.6 (10.6–54.0)	25.8	1.1
Minnesota	16	76,679	20,632	26.9	32.5 (9.2–71.9)	21.3	5.6
Maryland	12	130,809	35,102	26.8	29.5 (13.2–51.8)	17.9	8.9
District of Columbia	5	91,624	23,932	26.1	23.0 (5.6–46.0)	19.4	6.7
Mississippi	18	146,677	37,948	25.9	26.0 (11.7–48.7)	30.8	−4.9
Rhode Island	6	49,169	12,640	25.7	30.5 (17.1–40.6)	18.3	7.4
Virginia	23	166,179	41,099	24.7	29.9 (2.2–58.9)	21.6	3.1
Pennsylvania	29	204,291	49,920	24.4	30.4 (10.9–57.9)	23.7	0.7
Hawaii	12	52,740	12,843	24.4	21.3 (11.0–33.5)	14.4	10.0
Massachusetts	30	348,859	81,403	23.3	26.6 (5.4–85.5)	17.4	5.9
North Carolina	27	175,902	40,883	23.2	22.4 (0.4–41.0)	23.5	−0.3
Georgia	23	156,980	36,182	23.0	25.3 (6.0–48.8)	22.4	0.6
Delaware	3	23,055	5,301	23.0	23.1 (16.9–29.6)	20.6	2.4
Florida	38	385,604	88,308	22.9	26.0 (1.5–74.7)	18.5	4.4
Arizona	14	208,388	47,314	22.7	24.9 (13.3–70.9)	18.3	4.4
New York	51	759,384	165,743	21.8	29.3 (3.0–94.4)	17.9	3.9
New Jersey	18	186,291	39,153	21.0	26.5 (0.9–66.8)	16.7	4.3
Texas	61	516,650	104,219	20.2	21.2 (3.7–64.5)	18.8	1.4
Illinois	28	378,107	73,535	19.4	22.9 (4.5–53.0)	19.7	−0.3
Vermont	7	74,147	14,218	19.2	22.7 (8.4–41.5)	18.9	0.3
California	95	1,270,742	228,999	18.0	20.2 (4.3–78.8)	13.6	4.4
Utah	10	50,669	7,766	15.3	20.0 (2.9–48.9)	12.1	3.2

Abbreviations: BRFSS, Behavioral Risk Factor Surveillance System; FQHC, federally qualified health center.

a 967 FQHCs, which use the electronic health record to report clinical data, were included in the analysis.

b Number of patients that use tobacco divided by the number of total patients.

c Data from 2013 BRFSS.

d Difference in rate of tobacco use between patients at FQHCs and population.

Except for 5 states, state-level prevalence of tobacco use in FQHCs was higher than the BRFSS national average (Table). FQHC tobacco use prevalence and differences between FQHC and state-level estimates are displayed in the Figure.

**Figure:**
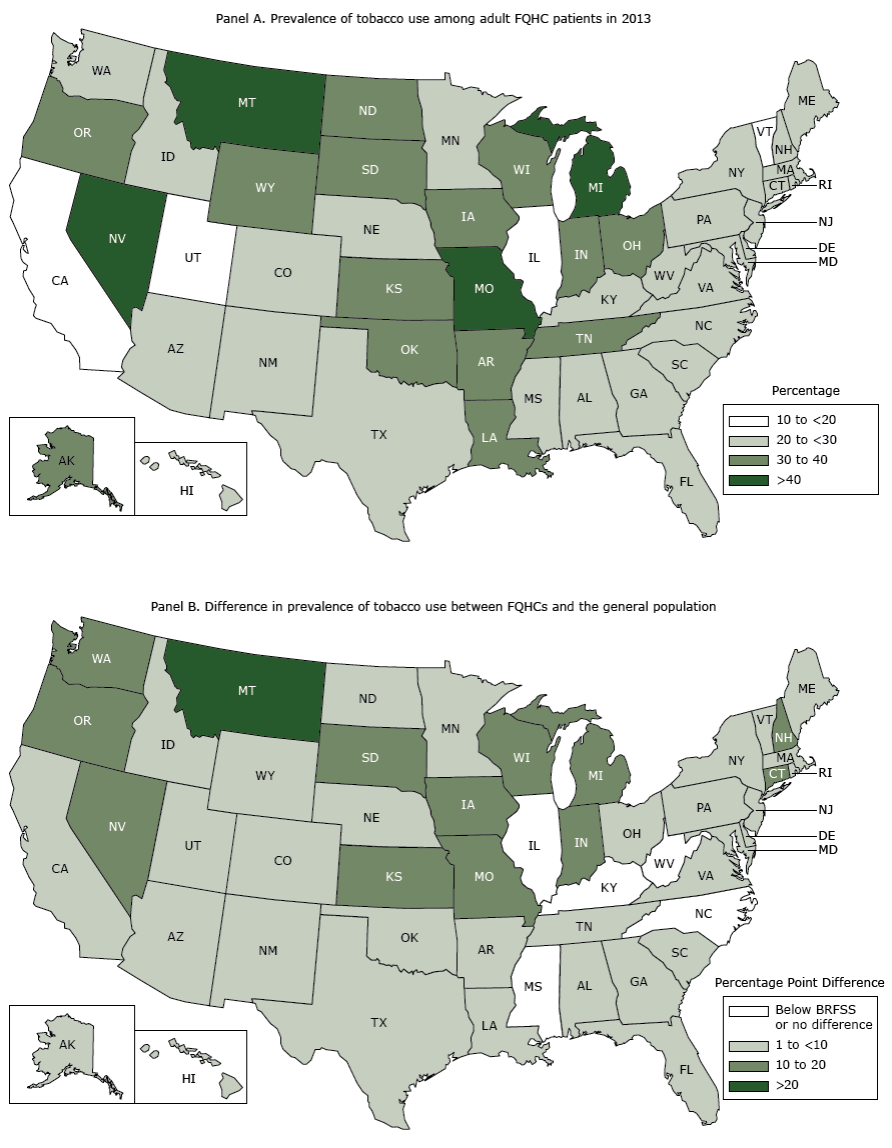
Federally qualified health center (FQHC) tobacco use prevalence and differences between FQHC and state-level estimates. Panel A shows the US prevalence of tobacco use among adult FQHC patients in 2013; panel B shows the differences in prevalence of tobacco use between FQHCs and the general population. Sources: Uniform Data System, 2013 (Panels A and B), and Behavioral Risk Factor Surveillance System, 2013 (Panel B).

Prevalence of tobacco use among individual FQHCs varied widely, even within states. Fifty-four percent of FQHCs had a tobacco prevalence greater than 30%; 63 FQHCs had tobacco use rates higher than 50%.

## Discussion

Our study is the first national assessment of the prevalence of tobacco use across FQHCs; previous reports focused on patient samples (9) or delivery of services among a subgroup of FQHCs (10). We found that in 2013 tobacco use among FQHC populations was considerably higher than for the general US population. Although the finding was not surprising, this report quantifies this difference for the first time. A second notable finding was the wide range of tobacco use and high prevalence of tobacco use in some FQHCs, particularly in sites where more than half of adult patients use tobacco. Caring for patients in an environment where the prevalence of tobacco use is high poses substantial challenges and may require additional investment of resources to successfully offer tobacco cessation.

Assessing tobacco use rates is an important first step to targeting opportunities for intervention and quality improvement (4). Implementing clinical interventions and decision support tools to effectively act on EHR-documented tobacco use to support delivery of tobacco treatment has emerged as a national priority, especially in low-income settings (3–6).

This study has 2 main limitations. First, UDS data are collected for administrative purposes rather than for research; we cannot verify outlier values or dictate how variables are documented. Conversely, BRFSS data collection procedures are standardized but rely on self-report. In an effort to report the most robust data possible, we limited analyses to FQHCs that generated UDS quality elements using an EHR so that estimates are based on the patient population rather than a random sample of manually abstracted records. To ensure data reliability, we examined 2 prior years of UDS reporting for the top and bottom 5% of FQHC tobacco use values; 2013 reporting prevalence was similar in all cases. Second, BRFSS is able to separate data on rates of combustible tobacco use and rates of smokeless tobacco use and in 2013 reported all tobacco use at 20.6%, combustible at 19.4%, and smokeless tobacco at 4% (7). However, UDS data combine all tobacco use (combustible and smokeless), limiting our ability to report tobacco use separately.

Recommendations by the US Preventive Services Task Force to offer annual lung cancer screening using low-dose computed tomography (LDCT) to long-term smokers older than 55 years will significantly affect FQHCs caring for older adults (11). Although the UDS cannot provide information on the number of individuals eligible for lung cancer screening (data on age and pack-year history are lacking), given tobacco user prevalence, the effort to implement the LDCT scans in FQHCs is substantial and will require an evaluation of costs and approaches to integrating smoking cessation (12). Understanding more about how FQHC clinicians, staff, and patients are addressing tobacco use — and how they plan to address lung cancer screening — is essential for guiding efforts to implement systems- and evidence-based practices to promote tobacco cessation and offer lung cancer screening to eligible patients.
